# Fathers at risk: Effects of threatened and actual preterm birth on the course of paternal peripartum depression and anxiety

**DOI:** 10.1371/journal.pone.0356746

**Published:** 2026-09-09

**Authors:** Valia Pernidaki, Karin Schermelleh-Engel, Sarah Sommerlad, Frank Louwen, Silvia Oddo-Sommerfeld

**Affiliations:** 1 Goethe-University Frankfurt, Institute of Psychology, Frankfurt am Main, Germany; 2 Goethe-University Frankfurt, University Hospital Frankfurt, Department of Obstetrics and Perinatal Medicine, Frankfurt am Main, Germany; Norwegian Institute of Public Health: Folkehelseinstituttet, NORWAY

## Abstract

**Background:**

While it is established that preterm birth (PB) increases the risk of psychological distress in fathers postpartum, the impact of a threatened PB on peripartum depressive and anxiety symptoms, as well as their course over time, remains insufficiently studied.

**Methods:**

We assessed 116 men during their partner’s pregnancy (T1) and 6 weeks postpartum (T2), using the Edinburgh Postnatal Depression Scale and the State-Trait Anxiety-Depression Inventory. Participants were categorized into a control group (CG) with term birth and two risk groups based on PB occurrence: 33 men with partners at risk of PB who had a term birth (RG-TB), and 38 men with partners at risk of PB who experienced a PB (RG-PB). Psychological distress was analyzed using multi-sample latent growth curve modeling.

**Results:**

During pregnancy, psychological distress varied across groups: fathers in the RG-PB had elevated levels of depression and anxiety compared to the CG, while the RG-TB presented only weak evidence of heightened depressive but not anxiety symptoms. Notably, both risk groups had higher rates of elevated depressive symptoms than the CG. Postpartum, depressive symptoms did not differ between groups. However, fathers in the RG-PB had higher anxiety scores than fathers in the CG. Although trajectory analyses revealed no distinct group-specific patterns, the RG-TB consistently showed intermediate levels of distress between RG-PB and CG.

**Conclusion:**

Threatened PB is associated with increased prenatal psychological distress in fathers, regardless of birth outcome. The combination of threatened and actual PB is linked to persistently elevated anxiety symptoms postpartum.

**Clinical relevance:**

Threatened PB identifies fathers at risk for psychological distress, warranting antenatal and follow-up screening, particularly due to persistent anxiety after PB. Fathers experiencing threatened PB followed by term birth remain vulnerable, as antenatal and postpartum emotional distress suggest the need for continued postpartum screening.

**Trial registration:**

ClinicalTrials.gov: NCT01974531

## Introduction

Parenthood can bring profound meaning to parents’ lives, enhance their well-being, and fill them with joy [e.g., 1, 2]. However, fathers may face particular challenges following preterm birth (PB). PB is defined as birth occurring before 37 completed weeks of gestation, with subgroups including extremely preterm (<28 weeks), very preterm (28– < 32 weeks), and moderate or late preterm (32– < 37 weeks) [3,4]. In 2020, approximately 13.4 million infants worldwide were born preterm, of whom about 4.2% were born before 28 weeks of gestation [5], with consequences for their survival, health, and development [6,7]. In Germany, approximately 8% of infants are born preterm, including about 0.7% extremely preterm infants [8,9].

PB remains the leading cause of neonatal morbidity and mortality [5]. The associated risks increase markedly with decreasing gestational age and birth weight. Extremely preterm infants (<28 weeks of gestation) have the highest rates of complications, including respiratory distress, intraventricular hemorrhage, cognitive impairment, and long-term neurodevelopmental disability [10].

Conversely, the risk of death, severe complications, and long-term impairment decreases with each additional week of gestation and each 150–250 g increase in birth weight [11]. Among infants born between 26 and 32 weeks of gestation, the duration of neonatal hospitalization decreases with advancing gestational age, with each additional week of pregnancy reducing hospital stay by at least eight days [11]. Very preterm infants are often hospitalized until approximately term-equivalent age (ca. 40 weeks post-menstrual age), although many infants, especially those born at later gestational ages, are discharged earlier [12].

For fathers, the medical vulnerability of their infants and the stressful neonatal intensive care environment can complicate bonding and elevate psychological strain, including guilt, uncertainty, and fear of contact [13–16]. Research indicates that PB is associated with increased paternal depression and anxiety. Antepartum depression affects about 9.7–19.5% of expectant fathers during the third trimester of pregnancy [17,18], with higher rates observed when their partners experience pregnancy complications requiring hospitalization. Following PB, about 17% of fathers develop postpartum depression (PPD) [19], compared to 8.75% prevalence typically reported after term birth (TB) [20].

Findings on paternal depressive symptoms following PB are mixed. Some studies report persistently elevated distress levels among fathers of preterm infants, whereas others suggest that depressive symptoms peak shortly after birth but then decrease rapidly [17,21–23]. Evidence for anxiety symptoms is more consistent. Approximately 12–25% of fathers report antepartum anxiety symptoms [17,18], and around 18% experience postpartum anxiety [19]. Risk factors include low income and education, limited social and co-parenting support, and maternal psychological distress [24]. Studies consistently show higher anxiety levels among fathers following PB compared with TB across multiple time points during the first postpartum year [17,21,25–27].

Few studies have examined paternal psychological distress prior to a PB. Studies investigating fathers during pregnancies at risk for PB are scarce and typically assess mental health only postpartum. For example, Petersen and Quinlivan [17] found no significant differences in clinically relevant antepartum depressive or anxiety symptoms between fathers of preterm and term infants. Similarly, Helle et al. [27,28] reported that an actual PB—but not a high-risk pregnancy—predicted paternal postpartum distress. However, these studies did not examine fathers’ mental health trajectories across the peripartum period.

Research examining the course of paternal mental health following PB remains limited. Studies focusing primarily on the postpartum period suggest heterogeneous trajectories. Some fathers show declining depressive symptoms during the first year after birth [25,29,30], whereas others experience persistently elevated symptoms [31,32]. A similar pattern has been reported for anxiety symptoms. Although postpartum anxiety levels may decline over time, they often do not fully return to baseline levels [25]. Persistent anxiety symptoms following PB have been observed from the third trimester through the early postpartum weeks [17] and up to one year after birth [25,32]. McMahon et al. [32], for instance, reported that approximately 50% of fathers experienced moderate levels of anxiety, while the other half experienced low but persistent anxiety throughout the first year postpartum.

Despite growing interest in paternal mental health following PB, important gaps remain. Little is known about how fathers’ mental health changes from pregnancy to the postpartum period, particularly when contrasting those facing a threat of PB versus those experiencing an actual PB. Comparing these groups may help disentangle the psychological impact of the prolonged threat of PB from the consequences of the birth outcome itself.

### Aims of the study

This study investigated paternal depressive and anxiety symptoms in relation to both the risk of and the experience of PB, conceptualizing PB not only as a birth event but as a stressor beginning during pregnancy. Specifically, we differentiated between two risk groups (RG) of fathers whose partners faced the risk of PB but had different birth outcomes: fathers whose infants were born preterm (RG-PB) and those whose infants were born at term (RG-TB). A control group (CG) consisted of fathers whose partners had no risk of PB and gave birth at term. Fathers’ depressive and anxiety symptoms were assessed during the late second or third trimester of pregnancy (T1) and again six weeks postpartum (T2).

The first aim was to investigate whether fathers in the RG-PB already differed from fathers in the RG-TB in their levels of psychological distress before birth (T1). We hypothesized that fathers in both risk groups would report higher levels of depressive and anxiety symptoms than fathers in the control group. Furthermore, because fathers in the RG-PB were assessed approximately two weeks earlier in pregnancy, when the threat of preterm birth was expected to be more imminent, we hypothesized that they would report higher levels of psychological distress than fathers in the RG-TB.

The second aim was to examine whether trajectories of depressive and anxiety symptoms differed between the three groups across the peripartum period. We hypothesized that trajectories of depressive and anxiety symptoms spanning the time from pregnancy to six weeks postpartum would differ between the three groups. However, fathers in the RG-PB were expected to report the highest levels of depressive and anxiety symptoms at T2 compared to RG-TB and CG, reflecting the combined impact of the prolonged threat of preterm birth and the subsequent experience of an actual preterm birth.

## Methods

### Participants and procedure

A total of 118 men were recruited at Frankfurt University Hospital as part of a comprehensive, longitudinal study investigating the effects of (threatened) preterm birth (PB) on the mental health of (expectant) parents. The research project examines a wide range of outcomes related to parental psychological distress (e.g., depression, anxiety, stress, and burnout), along with relevant protective factors (e.g., social support, partnership-related factors, optimism) from pregnancy up to two years after birth. Psychological data were self-reported, whereas medical data were obtained from the hospital’s clinical database. Recruitment of the men took place between 03/02/2013 and 08/15/2017. Fathers to be were recruited in person or received questionnaires via their partners. The first survey was completed by the men during the late second or third trimester of their partners’ pregnancies (T1), when the mothers to be were either registered for childbirth (control group, CG) or hospitalized due to medical complications (risk group, RG). The second survey was mailed six weeks postpartum (T2), and follow-up reminders were sent for un-returned questionnaires. Fathers were assigned to the RG based on their pregnant partners’ admission to the obstetric department due to pregnancy-related complications associated with PB, such as cervical insufficiency (CI), premature uterine contractions (PUC), preterm premature rupture of membranes (PPROM), and vaginal bleeding. The CG consisted of fathers whose partners were not at risk for PB and came to our department as outpatients to register for childbirth. Eligibility criteria for women were a minimum age of 18 years, a minimum gestational week of 24, and good knowledge of the German language. Exclusion criteria included a history of severe psychiatric disorders (e.g., schizophrenia) or neurological disorders, and substance abuse (excluding nicotine). Two men were excluded from the analyses because they reported an acute anxiety disorder at T1. Ethical approval was obtained from the university’s institutional review board, No. 391/12. Participation was voluntary, and all participants gave informed written consent prior to their inclusion in the study. Part of our study findings on women’s mental health in association with risk of PB has been previously published [33].

In total, *N* = 116 men participated in this study at the two measurement time points. The CG consists of *n*_*T1*_ = 45 men at T1 and *n*_*T2*_ = 41 men at T2, whose partners gave birth to healthy term babies. The RG comprises *n* = 71 men whose partners were hospitalized during pregnancy due to the risk of a PB. Within the RG, *n*_*T1*_ = 33 and *n*_*T2*_ = 28 men had partners who gave birth at full term (RG-TB), while *n*_*T1*_ = 38 and *n*_*T2*_ = 29 fathers had partners who experienced an actual PB (RG-PB).

### Instruments

The German version of the Edinburgh Postnatal Depression Scale (EPDS) [34] by Bergant et al. [35] was used to measure men’s depression at two time points. The EPDS consists of 10 items that capture mood over the past week, excluding typical somatic symptoms of depression that are common in the peripartum period. Each item is rated on a four-point rating scale ranging from 0 to 3, with the total score ranging from 0 to 30. A commonly used cut-off score for men of ≥10 was applied to indicate minor depression symptoms [36]. The EPDS has been validated for men demonstrating acceptable validity and high internal consistency values [36]. McDonald’s omega [37] in this study was sufficiently high with ω = .82 at time point 1 and ω = .86 at time point 2.

The State-Trait Anxiety-Depression Inventory (STADI-S) [38,39] is a 40-item self-report questionnaire that enables a differentiation between depression and anxiety. It captures depression and anxiety as stable dispositions (traits) as well as unstable situation-dependent moods (states). In this study, we used the 10-item anxiety state scale (STADI-S). Each item is scored on a rating scale ranging from 1 (*not at all*) to 4 (*very much*) with a maximum sum score of 40 for the scale. Scores indicating enhanced state anxiety were defined using elevated T-values in the STADI-S as suggested by the authors, corresponding to sum scores for each subscale ≥ 22 [35]. McDonald’s omega of the state anxiety scale was sufficiently high with ω = .86 at time point 1 and ω = .85 at time point 2.

### Statistical analysis

Descriptive statistics and correlation coefficients (Pearson’s *r* and Spearman’s *ρ*) were calculated using SPSS, version 29 [40]. Multi-group latent growth curve modeling (LGCM), a statistical technique within the framework of structural equation modeling (SEM), was used to model change over time simultaneously for the three groups: RG-PB, RG-TB, and CG. As the variables were not normally distributed, the robust maximum likelihood estimator (MLR) of the M*plus* program, version 8 [41], was used for parameter estimation. Another advantage of MLR is that all available data from participants were used despite some missing values at the second measurement time point. To test for possible covariates, we first performed two separate linear regression analyses with depressive and anxiety symptoms at T1 as the dependent variables.

Model fit was evaluated using the Yuan-Bentler corrected χ^2^-value as well as the descriptive fit criteria root mean square error of approximation (RMSEA) and comparative fit index (CFI) [cf. 42, 43]. Good model fit was indicated by a non-significant robust χ^2^-value, RMSEA ≤ .05, and CFI ≥ .97.

**Fig 1:**
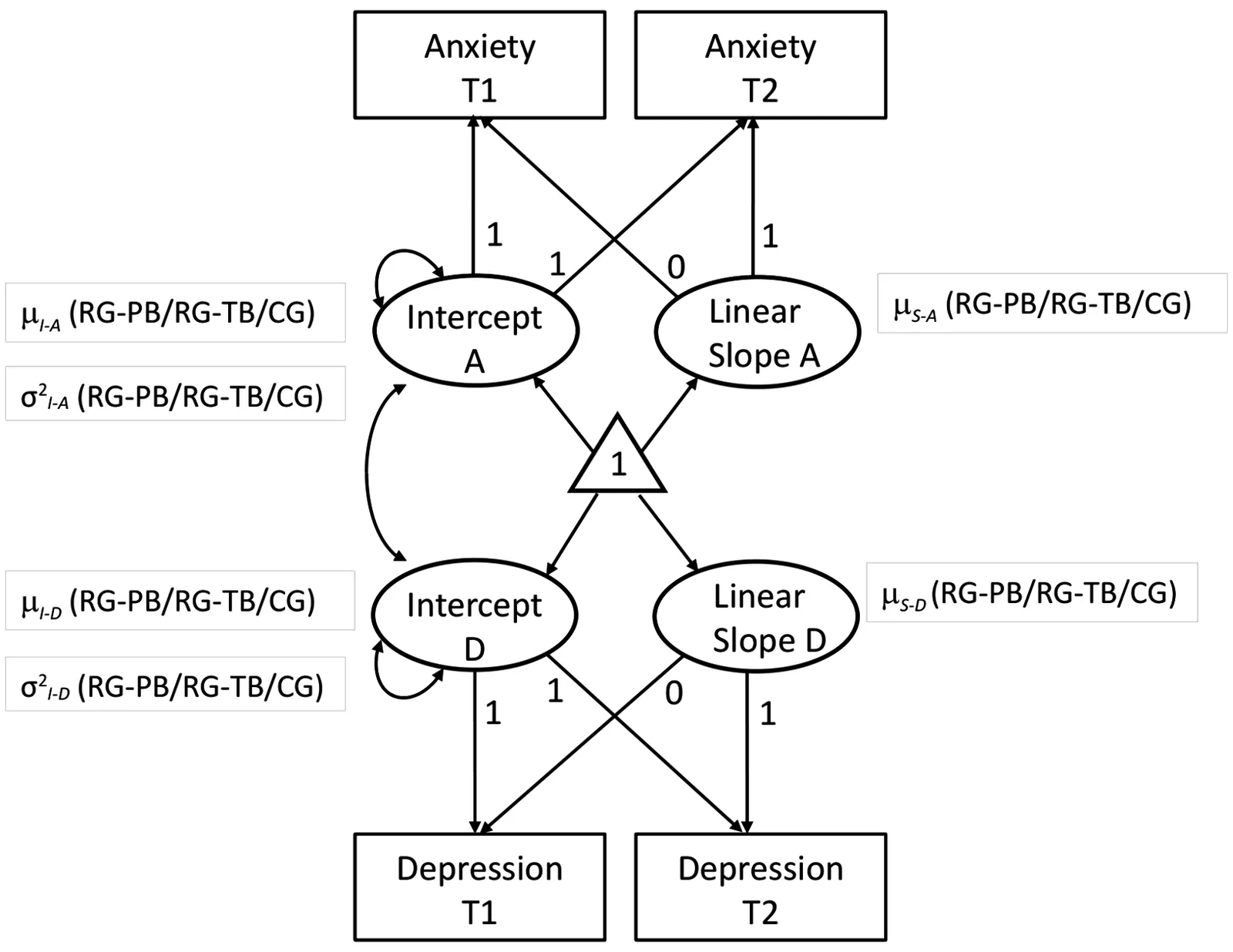
Multi-group LGCM of perinatal anxiety and depression for CG, RG-TB, and RG-PB. T1 = late 2^nd^ or 3^rd^ pregnancy trimester, T2 = six weeks postpartum; CG = control group, RG-TB = risk group and term birth, RG-PB = risk group and preterm birth; the triangle denotes the latent mean structure; squares represent observed variables, and circles latent variables, i.e., intercepts and linear slopes for anxiety (A) and depression (D). Residual variances were constrained to be equal across groups. Due to the two-wave design, slope variances and covariances had to be fixed to zero. The latent means of the anxiety and depression intercept and slope factors are denoted by μ_*I-A*_, μ_*I-D*_, μ_*S-A*_, and μ_*S-D*_, the latent intercept variances by σ^2^_*I-D*_ and σ^2^_*I-A*_.

Using multi-group LGCM, we investigated the change of depression and anxiety scores simultaneously in a common model with two time points (T1: late 2^nd^ or 3^rd^ trimester of pregnancy, T2: 6 weeks postpartum) including two intercept factors and two slope factors (Fig 1). The multivariate approach enables to assess the covariance between intercepts of depression and anxiety. Residual variances for each construct, depression or anxiety, were set equal across all groups. Intercepts were allowed to vary between individuals to determine individual differences in each group. Due to only two measurement time points in this study, only linear trajectories could be estimated. The variance of both depression and anxiety slope factors had to be fixed to zero, while the intercept factors for both outcome variables were allowed to correlate. To investigate parameter differences across the three groups, model constraints were specified and tested. As we had expectations concerning the order of mean values for group differences and time points, we applied one-tailed significance tests at *p* ≤ .05.

## Results

### Sample characteristics

Socio-demographic and pregnancy-related sample characteristics are listed in Table 1. Men in the CG were recruited significantly later in pregnancy compared to men in the RG-TB and RG-PB, while men in the RG-PB were recruited significantly earlier than those in the RG-TB. No significant differences were found between the three groups in socio-demographic variables, except for past mental illness. Men in the RG-TB had a higher rate of past mental illness compared to men in the RG-PB and CG. Women in the RG-PB had significantly higher rates of PB-related obstetric complications than women in the RG-TB with regard to CI and PUC, but not with regard to vaginal bleeding and PPROM (see Table 1). Respiratory-distress syndrome prophylaxis was more frequent in the RG-PB (71.05%) than in the RG-TB (39.39%, χ^2^(1) = 7.20, *p* = .007). In addition to symptoms indicative of threatened PB, other pregnancy-related conditions were documented (not listed in Table 1), including gestational diabetes (CG: 8.89%; RG-TB: 15.63%; RG-PB: 13.51%; *LR*(2) = 0.89, *p* = .642), and pre-eclampsia (RG-PB: 11.54%; RG-TB: 11.11%; χ^2^(2) = 0.00, *p* = .961). Mean BMI was 23.18 (*SD* = 2.82) in the RG-PB, 23.81 in the RG-TB (*SD* = 2.98) and 23.29 (*SD* = 2.81) in the CG, *F*(2, 57) = 0.23, *p* = .799.

**Table 1:** Socio-demographic and pregnancy-related characteristics of study participants by subgroup.

	CG (*N* = 45)	RG-TB (*N* = 33)	RG-PB (*N* = 38)	Significance
*Socio-demographic Characteristics*				
Age (*M*; *SD*)	35.86; 6.40	36.13; 5.20	33.61; 4.51	*F*(2, 111) = 2.37, *p* = .098 (a)
Nationality				LR(2) = 0.04, *p* = .981 (b)
German	91.11%	90.91%	92.11%	
Other	8.89%	9.09%	7.89%	
Education				χ^2^(2) = 0.85, *p* =.654(c)
≤ 10 years of schooling	36.36%	34.38%	27.03%	
> 10 years of schooling	63.64%	65.62%	72.97%	
Relationship duration				LR(4) = 0.37, *p* = .985 (b)
> 1 year	13.64%	15.15%	11.11%	
> 3 years	18.18%	15.15%	16.67%	
> 5 years	68.18%	69.70%	72.22%	
Children at T1				χ^2^(2) = 3.26, *p* = .196 (c)
Yes	31.11%	18.18%	15.79%	
No	68.89%	81.82%	84.21%	
Past mental illness				LR(2) = 13.40, *p* = .001 (b)
Yes	4.44% ^a^	31.25% ^a,b^	5.26% ^b^	
No	95.56%	68.75%	94.74%	
*Pregnancy-related Characteristics*				
Women’s gestational age in weeks (*M*; *SD*)				
when recruited	35.11 ^a,b^; 1.09	30.64 ^a,c^; 3.31	28.62 ^b,c^; 3.41	*F*(2, 112) = 62.18, *p* < .001 (a)
at birth	39.60 ^a^; 1.16	38.61^b^; 1.52	32.21 ^a,b^; 3.13	*F*(2, 113) = 143.79, *p* < .001 (a)
Planned pregnancy				χ^2^(2) = 1.67*, p* = .435 (c)
Yes	82.22%	84.37%	91.89%	
No	17.78%	15.63%	8.11%	
Women’s mode of birth				LR(4) = 7.92, *p* = .094 (b)
Spontaneous vaginal	54.76%	53.33%	32.36%	
Caesarean section	26.19%	36.67%	55.88%	
Instrumental vaginal	19.05%	10.00%	11.76%	
*PB-related obstetric symptoms*				
CI				χ^2^(1) = 5.03, *p* = .025 (c)
Yes	---	40.62%	67.57%	
No	---	59.38%	32.43%	
PUC				χ^2^(1) = 4.23, *p* = .040 (c)
Yes	---	38.46%	66.67%	
No	---	61.54%	33.33%	
PPROM				
Yes	---	18.75%	36.11%	χ^2^(1) = 2.54, *p* = .111 (c)
No	---	81.25%	63.89%	
Vaginal bleeding				
Yes	---	25%	9.09%	χ^2^(1) = 2.02, *p* = .155 (c)
No	---	75%	90.01%	

*Note.* PB = Preterm birth, CI = Cervical insufficiency, PPROM = premature rupture of membranes, PUC = premature uterine contraction. (a) Univariate ANOVA, (b) Likelihood Ratio Test, (c) Pearson’s χ^2^ Test.

a,b,c Same upper case letters of post-hoc-tests indicate a significant group difference at α = .05.

The infant sample comprised *N* = 126, including nine multiple births (6 twins, 2 triplets). For analyses, mean scores of multiples’ variables were used. Fathers in the RG-PB reported more often that their infant had been hospitalized in a neonatal intensive care unit compared to men in the RG-TB (73.68% vs. 21.88%, χ²(1) = 18.65, *p* < .001). Fathers in the RG-PB reported higher depressive symptoms when their infants were hospitalized in the neonatal intensive care unit after childbirth (*M* = 5.86, *SD* = 1.09) compared to those whose infants were not hospitalized (*M* = 2.25, *SD* = 1.83), *t*(27) = −2.84, *p* = .004. At T2, 12 men reported that their infants were still hospitalized; of these, only one belonged to the RG-TB. Infants in the three groups differed significantly in birth weight, *F*(2, 67.10) = 50.08, *p* < .001. Babies in the RG-PB (*M* = 1.933 kg, *SD* = 0.754 kg) had lower birth weights than those in the RG-TB (*M* = 3.286 kg, *SD* = 0.508 kg, *p* < .001) and the CG (*M* = 3.390 kg, *SD* = 0.561 kg, *p* = .001). No significant difference was observed between the RG-TB and CG. APGAR scores at 1 minute differed significantly between groups, *F*(2, 56.49) = 7.78, *p* = .001. Infants in the RG-PB had lower scores (*M* = 7.52, *SD* = 1.76) than those in the RG-TB (*M* = 8.69, *SD* = 0.74) and the CG (*M* = 8.78, *SD* = 0.67), all *p*s < .001. No significant difference was observed between the RG-TB and CG.

### Rates of elevated depression and anxiety scores

Rates of elevated paternal depression and anxiety scores are based on a total sample size of *N* = 116 with varying amounts of missing values (a maximum of 10 for anxiety at T2 in the RG-PB) (Table 2).

**Table 2:** Rates of elevated depressive and anxiety symptoms in the late 2^nd^ or 3^rd^ trimester of pregnancy (T1) and six weeks postpartum (T2).

	Time point	CG (*N* = 45)	RG-TB (*N* = 33)	RG-PB (*N* = 38)
Depression (EPDS ≥ 10)	T1	4 (9.09%)	9 (28.13%)	13 (36.11%)
	T2	2 (4.88%)	5 (17.24%)	2 (6.90%)
Anxiety (STADI-S ≥ 22)	T1	16 (35.56%)	11 (34.38%)	20 (54.05%)
	T2	4 (10.0%)	4 (14.29%)	7 (25.0%)

*Note.* Entries are listed as *N* (%)

The highest rate of elevated depressive symptoms was found in the RG-PB at T1 (36.11%), followed by the RG-TB and the CG. Men in the RG-PB were 5.65 times more likely to score above the EPDS cut-off compared to men in the CG (*b* = 1.73, Wald χ^*2*^(1) = 7.59, *p* = .006). Participants in the RG-TB were 3.91 times more likely to experience elevated depression scores at T1 compared to those in the CG (*b* = 1.36, Wald χ²(1) = 4.33, *p* = .037). At T2, the RG-TB had the highest percentage of fathers screening positive for depressive symptoms (17.24%), followed by RG-PB (6.90%) and CG (4.88%), though the differences did not reach statistical significance across groups. Men in the RG-PB exhibited the highest rates of elevated anxiety scores at both T1 (54.05%) and T2 (25.0%). However, no significant differences in anxiety rates existed across the groups.

### Relationships between socio-demographic and clinical variables

Correlation coefficients for socio-demographic and clinical variables are presented in Table 3, including maternal obstetric and mental health variables. Gestational age at T1 and at birth showed small positive associations with vaginal delivery and negative associations with cesarean delivery. Both variables were negatively correlated with paternal and maternal psychological distress at T1 (*r*s = −.24 to −.45). Past mental illness was not significantly associated with any of the study variables. Paternal anxiety and depression scores were significantly correlated across the two time points, indicating temporal stability. The association between paternal and maternal depression at T1 was not significant, whereas maternal and paternal anxiety levels at T1 were modestly correlated (*r* = .27).

**Table 3:** Correlations between socio-demographic, obstetric, and clinical variables (*n*_*T1*_ = 116; *n*_*T2*_ = 88).

	1	2	3	4	5	6	7	8	9	10	11	12	13	14	15
1. Age	1														
2. Education	−.08	1													
3. Gestational age at T1	.05	−.18	1												
4. Gestational age at birth	.18	−.13	.63**	1											
5. Relationship duration	−.07	−.09	.01	−.06	1										
6. Children at T1	.42**	−.13	.16	.09	.16	1									
7. Past mental illness	.15	.04	−.06	.16	−.10	−.01	1								
8. Planned pregnancy	−.13	−.02	−.15	−.12	.32**	−.08	−.01	1							
9. Mode of birth (vaginal)	−.16	.09	.23*	.22*	.09	.05	−.04	.03	1						
10. Mode of birth (cesarean)	.13	−.08	−.21*	−.28**	−.01	.01	.08	.01	−.75**	1					
11. Depression T1	−.01	.02	−.28**	−.26**	.08	.02	.11	.18	−.17	.04	1				
12. Depression T2	−.17	−.03	−.05	−.17	−.08	−.17	.03	.11	−.09	−.06	.19	1			
13. Anxiety T1	−.07	.00	−.24*	−.24*	.03	−.12	−.09	−.00	−.03	−.12	.55**	.32**	1		
14. Anxiety T2	−.07	−.04	−.15	−.19	−.07	−.19	.02	−.08	.03	−.22*	.45**	.31**	.75**	1	
15. Depression T1 (W)	.02	−.05	−.25**	−.31	.09	−.03	−.02	−.00	−.09	.03	.16	.11	.09	.18	1
16. Anxiety T1 (W)	−.22*	.01	−.39**	**−**.45**	.12	−.15	−.12	.19*	.00	.01	.24*	.00	27**	.24*	.52**

*Note*. W = Women. Dichotomous variables coded as 0 = no, 1 = yes are the following: children at T1, past mental illness, planned pregnancy, mode of birth (vaginal,) and mode of birth (cesarean). Education: 0: ≤ 10 years of schooling 1: > 10 years of schooling. Relationship duration: 0: > 1 year, 1: > 3 years, 2: > 5 years.

* *p* < .05; ** *p* < .01.

### Multi-group latent growth curve modeling

To examine the changes in the mean anxiety and depression trajectories in the three groups, a multi-group LGCM (Fig 1) was estimated. As socio-demographic and clinical variables (listed in Table 1) did not reach significance (all *p*s > .05), the LGCM was performed without including covariates.

### Within-group results

**Fig 2:**
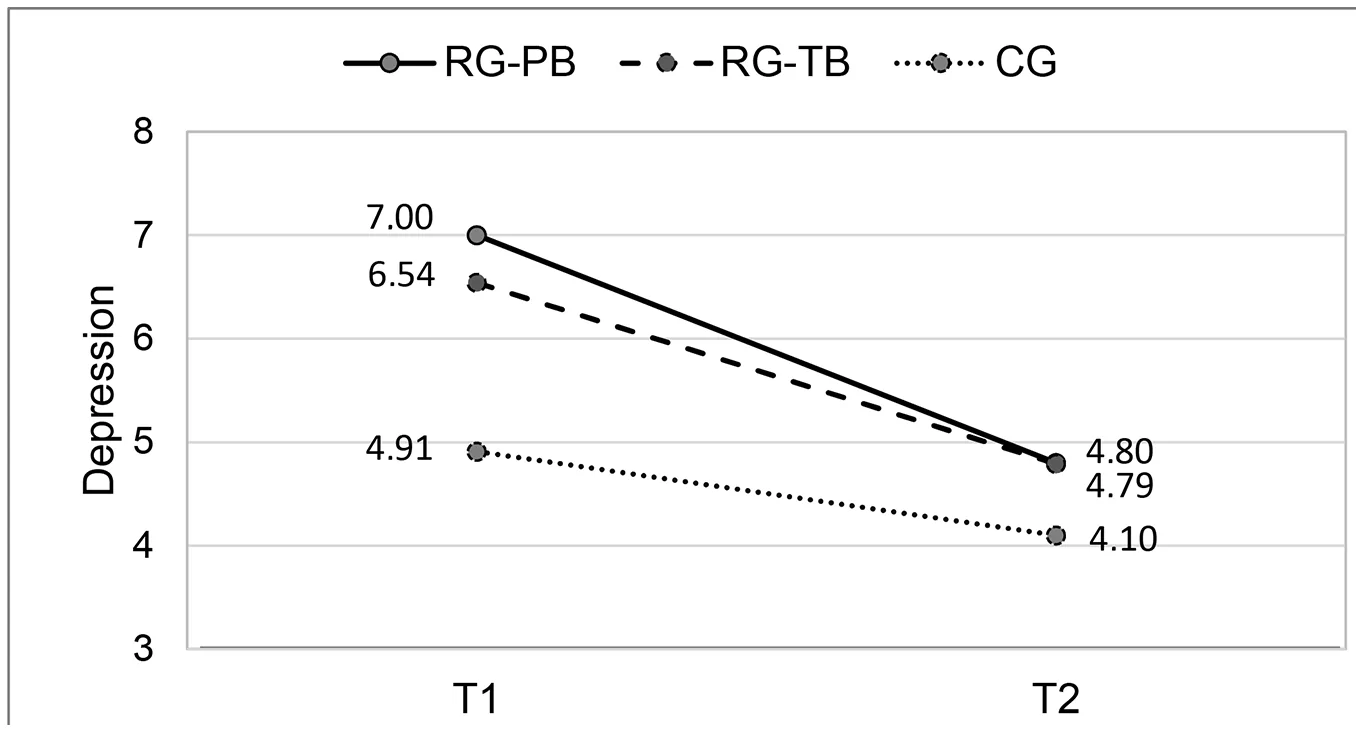
Mean depression scores for CG, RG-TB, and RG-PB. T1 = late 2^nd^ or 3^rd^ pregnancy trimester, T2 = six weeks postpartum.

**Fig 3:**
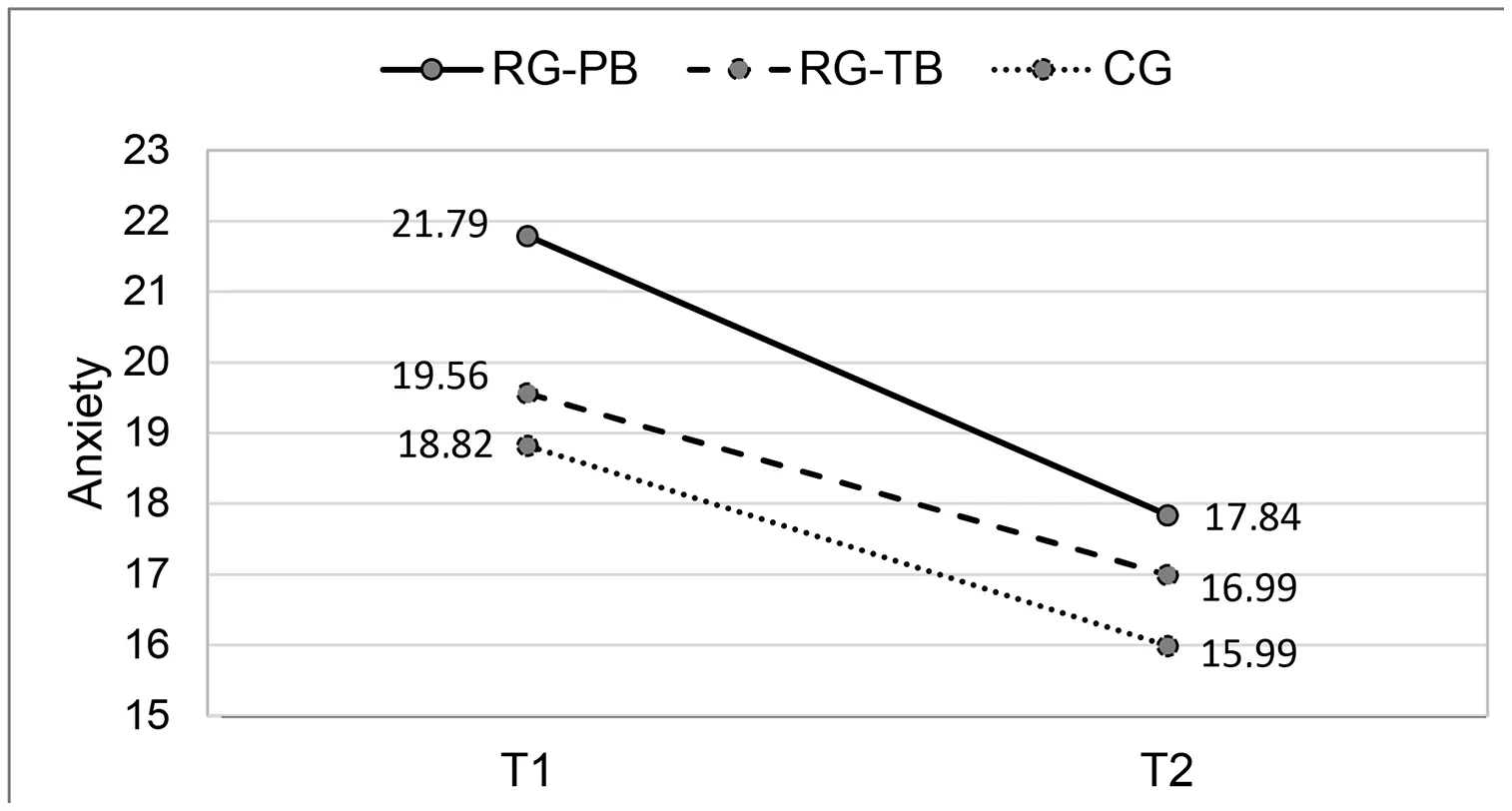
Mean anxiety scores for CG, RG-TB, and RG-PB. T1 = late 2^nd^ or 3^rd^ pregnancy trimester, T2 = six weeks postpartum.

Of all groups, RG-PB had the highest estimated mean depression values and the highest mean anxiety values at T1 (Table 4), followed by the RG-TB and then the CG. The RG-PB also had the steepest decline over time (Figs 2 and 3). We observed significant variability in men’s depression scores at T1 in the RG-TB, but less variability in the RG-PB, and even no significant variability in the CG. Within the three groups, intercept variances for anxiety were significant, suggesting substantial individual variability around the means (Table 4). At T2, RG-PB had again the highest depression values (Fig 2) and anxiety values (Fig 3) followed by RG-TB and then by CG.

**Table 4:** Unstandardized parameter estimates for depression and anxiety based on the multivariate multi-sample latent growth curve model (*N* = 116).

	CG (*N* = 45)		RG-TB (*N* = 33)		RG-PB (*N* = 38)	
	*b*(SE)	*p*-value	*b*(SE)	*p*-value	*b*(SE)	*p*-value
*Mean Intercepts*						
Depression	4.91 (0.65)	.000	6.54 (0.80)	.000	7.00 (0.72)	.000
Anxiety	18.82 (0.76)	.000	19.56 (1.01)	.000	21.79 (0.76)	.000
Cov(D, A)	7.15 (2.45)	.004	11.57 (4.51)	.010	5.15 (2.34)	.028
*Mean Slopes*						
Depression	−0.81 (1.00)	.209	−1.75 (0.96)	.034	−2.20 (0.98)	.024
Anxiety	−2.83 (0.57)	.000	−2.57 (0.69)	.000	−3.95 (0.62)	.000
*Intercept Variances*						
Depression	4.77 (3.56)	.180	7.67 (4.22)	.069	5.39 (2.64)	.041
Anxiety	16.31 (2.99)	.000	22.90 (6.78)	.001	15.33 (2.73)	.000

*Note.* Model fit: χ^2^ = 17.539(16), *p* = .352, *RMSEA* = .050, *CFI* = .988, *SRMR* = .131. Depression was measured by EPDS, Anxiety by STADI – State Anxiety scale.

### Between-group results

#### Differences in the means of antepartum depression and anxiety.

At T1, mean depression scores were higher in the RG-PB than in the CG (*b* = 2.09, *SE* = 0.97, one-sided *p* = .016, 90% CI [0.50, 3.69]), providing support for the a priori directional hypothesis of higher psychological distress in the RG-PB. Depression scores were also estimated to be higher in the RG-TB than in the CG (*b* = 1.63, *SE* = 1.02). However, the 90% confidence interval included zero (90% CI [−0.05, 3.32]), indicating uncertainty regarding the magnitude of the difference. Consistent with the a priori directional hypothesis, the corresponding one-sided test yielded *p* = .055, suggesting that the data were compatible with a small positive effect but did not provide conclusive evidence for a group difference. There was no difference in depression scores between the two RGs (*b* = 0.46, *SE* = 1.07, one-sided *p* = .334, 90% CI [−1.31, 2.23]), indicating that the data did not support the hypothesis of higher prenatal depressive symptoms in the RG-PB than in the RG-TB.

For anxiety, scores were higher in the RG-PB than in the CG (*b* = 2.97, *SE* = 1.08, one-sided *p* = .003, 90% CI [1.94, 4.74]), providing support for our a priori directional hypothesis. In contrast, anxiety scores in the RG-TB were not higher than those in the CG (*b* = 0.74, *SE* = 1.27). The 90% confidence interval included zero (90% CI [−1.35, 2.83]), and the one-sided test yielded *p* = .279, providing no evidence for a group difference. Consistent with the a priori directional hypothesis, men in the RG-PB showed higher anxiety symptoms at t1 than men in the CG (*b* = 2.22, *SE* = 1.27, one-sided *p* = .04, 90% CI [0.14, 4.31]).

#### Differences in the means of postpartum depression and anxiety.

At T2, there was no evidence for differences in depressive symptoms across the three groups (all *p*s = .272–.497). However, fathers in the RG-PB had higher anxiety scores than those in the CG (*b* = 1.84, *SE* = 1.06, one-sided *p* = .041, 90% CI [0.10, 3.58]), providing support for the a priori directional hypothesis. In contrast, there was no difference in anxiety scores between the RG-TB and the CG (*b* = 1.00, *SE* = 1.15, *p* = .385, 95% CI [−1.25, 3.25]). Likewise, there was no difference in anxiety scores between the two risk groups (*b* = 0.84, *SE* = 1.24, one-sided *p* = .248, 90% CI [−1.19, 2.88]), indicating that the data did not support the hypothesis of higher postpartum anxiety in the RG-PB than in the RG-TB.

#### Differences between trajectories across groups.

All groups showed a significant decline in depression and anxiety mean slopes (all *p*s < .01) with the exception of paternal depression scores in the CG (*p* = .209) (Table 4). Across groups, no significant differences were identified in the mean trajectories of depressive symptoms from T1 to T2 (all *p*s > .05). Descriptively (Fig 2), men of the RG-PB showed the steepest decline, followed by the RG-TB and then the CG. For anxiety, the RG-PB showed again the steepest decline, followed by the CG and then the RG-TB (Fig 3). However, differences in the trajectories were not significant (all *p*s > .05). Generally, the results showed no support for distinct trajectories of the three groups, although the trajectory of the RG-TB group consistently fell between those of the other two groups for both depressive and anxiety symptoms.

### Follow-up analysis

Because men in the RG-TB were significantly more likely to report a past mental illness compared to men in the RG-PB and CG (see Table 1), we conducted an additional analysis including past mental illnesses as a covariate in the LGCM. Given the small number of cases in RG-PB and CG (only two men in these groups were affected), the LGCM with this covariate could not be estimated for these groups. Therefore, the analysis was restricted to the RG-TB. The results showed that past mental illness was not a significant covariate, neither for anxiety, *b* = 0.16, *SE* = 1.71, *p* = .926, nor for depressive symptoms, *b* = 1.30, *SE* = 1.57, *p* = .408. Therefore, past mental illness did not predict the latent anxiety and depression scores at T1.

## Discussion

### Principal findings

This study examined fathers whose partners were at risk for preterm birth (PB) and subsequently gave birth either to full-term babies (RG-TB) or to preterm babies (RG-PB). We investigated how the threat of PB influenced fathers’ antepartum psychological distress levels and its course from the late 2^nd^ or 3^rd^ trimester of their partners’ pregnancy to 6 weeks postpartum. Overall, our findings indicate that the threat of PB adversely affects the mental health of expectant fathers during the peripartum period. Specifically, at T1, fathers in the RG-PB reported the highest levels of distress, while anxiety symptoms appeared to be more pronounced than depressive symptoms. Although distress levels decreased by T2 in both risk groups, growth curve modeling did not reveal distinct trajectories across the three groups. However, the RG-TB showed intermediate levels of distress, falling between those of the other two groups for both depression and anxiety across both time points.

Focusing on depressive symptoms during pregnancy, fathers in the RG-PB showed more pronounced symptoms than those in the CG, while there was weak evidence that fathers in the RG-TB also reported higher symptom levels than those in the CG. Consistent with these findings, fathers in both risk groups reported higher rates of elevated antepartum depressive symptoms than fathers in the CG. The rates of elevated antepartum depression scores in our sample were higher than those observed in a recent study by Petersen and Quinlivan [17] (preterm: 9.7%, control: 8.4%). This discrepancy may be attributed to the different measurement instruments, as Petersen and Quinlivan [17] employed the HADS to assess depressive symptoms, while we used the EPDS.

We observed overall high rates of elevated anxiety symptoms during pregnancy across all groups (RG-PB: 54.05%; RG-TB: 34.38%; CG: 35.56%) with fathers in the RG-PB experiencing significantly higher levels than those in the CG. Furthermore, fathers in the RG-PB were more anxious than those in the RG-TB antepartum. One possible explanation is related to the timing of recruitment, which corresponded to the week of hospitalization because of the risk of PB. Partners of fathers in the RG-PB were admitted at 29 weeks of gestation, whereas those in the RG-TB were admitted 2 weeks later (at 31 weeks). The late second and third trimesters of pregnancy are an important time period for the fetus with rapid brain development and exponential increases in brain volume [44]. Since fetal development between 28 and 32 weeks is critical, the two-week difference in gestational age and therefore the heightened threat of PB may have contributed to increased anxiety scores. This is consistent with previous research indicating that levels of psychological distress rise with the severity of prematurity, with fathers of very premature infants often experiencing greater distress compared to fathers of full-term infants [45,46].

Our findings differ from those of Petersen and Quinlivan [17] who found no differences in antepartum depressive and anxiety symptoms between fathers of preterm and full-term infants. However, their study did not differentiate between RG-TB and RG-PB, so that the RG-TB fathers may be subsumed under the CG. Furthermore, in the present study, risk groups were defined based on distinct obstetric risk factors for the PB (e.g., cervical insufficiency, premature uterine contractions), rather than general high-risk pregnancies [27,28].

We also investigated changes in psychological distress from pregnancy to six weeks postpartum. Descriptively, depressive symptoms decreased most in the RG-PB, followed by the RG-TB and the CG. No significant change in depressive symptoms was observed in the CG. Molgora et al. [47] identified a subgroup of fathers who experienced a healthy pregnancy and childbirth, with stable and low depressive symptoms during the peripartum period. Other studies have reported different patterns, such as an increase in depressive symptoms from antepartum to postpartum [48] or a decrease six months postpartum [47].

In the present study, the observed decrease in depressive symptoms from T1 to T2 in both risk groups is consistent with previous findings showing improvements in fathers’ depression scores assessed between the first month and first year after a PB [25,29,30,49]. Notably, the highest rates of elevated depressive symptoms at T2 were observed in the RG-TB, although these differences were not statistically significant across groups. This finding suggests that fathers who experience a threatened PB may remain vulnerable even when a term birth occurs.

Anxiety symptoms also decreased significantly from T1 to T2 in all groups. Consistent with our findings, previous reviews suggest that anxiety levels may decrease from pregnancy to postpartum period, although many studies do not specify gestational timing at recruitment or child birth [24,50]. Although only a limited number of studies have examined the course of postpartum anxiety symptoms in fathers in relation to PB, available evidence suggests a reduction in symptoms over time [25,29]. Despite showing the greatest decline, fathers in the RG-PB continued to report higher anxiety symptoms than those in the CG at T2. This finding highlights the persistence of anxiety symptoms following PB and aligns with prior research [25]. Fathers are still often neglected in terms of interventions and support in the peripartum period, and our data underscore the need for further research and targeted support for both risk groups.

### Limitations and strengths

There are several limitations of this study. First, this study relied on self-report measures, which cannot replace a clinical diagnosis and may be prone to social desirability bias. The absence of structured clinical interviews may have led to an underestimation of paternal psychological distress, particularly among fathers in the RG-TB, whose symptom levels consistently fell between those of the CG and RG-PB. Clinician-administered interviews could help determine whether these intermediate symptom levels reflect clinically meaningful differences. Future studies should therefore incorporate structured clinical interviews to validate and extend our findings. Pérez et al. [51] emphasized the importance of using questionnaires beyond the EPDS to capture male-specific aspects of distress in research. Additionally, sample bias could not be ruled out, since male participants were recruited after their spouses had consented to take part in the study and generalization of results to the broader population may be therefore limited. Moreover, the participants were recruited from a single university hospital in Germany, which may further restrict the external validity of the findings.

A second limitation of our study is the relatively small sample size, which reduces statistical power and increases the risk of Type II errors. Consequently, true group differences in intercepts and slopes may not have been detected. Given the relatively small subgroup sizes, individual participants with atypical developmental trajectories may have had a disproportionate influence on the estimated group parameters. To improve model stability, we included equality constraints on the residual variances, thereby reducing the number of estimated parameters. Nevertheless, the results should be considered as exploratory and interpreted with caution. We recommend replicating our study using larger, multicenter cohorts and a greater number of measurement time points.

A third limitation is the different time points of recruitment, which may account for the differences found at the initial assessment during pregnancy and which may have further exacerbated the differences in emotional states among the three groups. Therefore, baseline (T1) group comparisons should be interpreted with caution. Moreover, eleven infants in the RG-PB remained hospitalized at the T2 assessment (6 weeks postpartum). Fathers of hospitalized infants reported higher depressive symptoms than those whose infants had been discharged. Although ongoing hospitalization and medical uncertainty may have contributed to increased paternal distress, this relationship could not be examined in detail due to the modest sample size. Additionally, some mothers gave birth outside the study site, and together with attrition at T2, this limited the availability of hospitalization and infant morbidity data. Future research should explore the extent to which hospitalization duration and infant illness severity contribute to mental health outcomes among fathers of preterm infants.

Fourth, longitudinal designs are essential for understanding changes in parental well-being during the peripartum period, as noted, among others, by Paulson et al. and Philpott et al. [52,53]. However, our study included only two assessment time points (during pregnancy and at six weeks postpartum) and therefore provides only limited insight into changes in paternal psychological distress across the perinatal period. With only two measurement time points, we were able to model only linear change over time; nonlinear patterns of change and more complex symptom trajectories could not be examined. Moreover, individual differences in developmental trajectories, which may have important implications for clinical practice, could not be estimated because slope variances were fixed. Consequently, the estimated symptom trajectories should be regarded as simplified representations of psychological change over the perinatal period and interpreted with caution. Including additional measurement points, such as multiple assessments during pregnancy and the postpartum period, would enable a more nuanced characterization of symptom trajectories. This is particularly relevant because studies with more frequent assessments have demonstrated that changes in symptom levels over time, rather than stable symptom levels alone, may be associated with adverse birth outcomes [54].

Fifth, evidence suggests that the severity of prematurity correlates with greater psychological strain in fathers [26,30,45,46], although this association was not explored in the present study. Given the sample size and model complexity, including additional covariates in the LGCM would likely have reduced statistical power and compromised model stability. Several socio-economic factors are known to influence parental mental health, although less is known about their effects on fathers. Protective factors include higher education, stable relationships, and first-time fatherhood, whereas lower socioeconomic status, limited social support, poor relationship quality, additional caregiving responsibilities, and maternal psychological distress have been associated with poorer paternal mental health [24,29,48,55]. In our sample, most fathers had a high level of education, were in long-term relationships (>5 years), and were first-time fathers, which may have been protective. Apart from maternal anxiety and depression at T1, which showed only small associations with fathers’ mental health, we did not assess other potential risk factors. Future research should examine the combined influence of socio-economic and psychosocial risk factors on fathers’ mental health, particularly among men facing competing financial and caregiving demands following threatened or actual PB.

Sixth, it should be noted that maternal psychological distress was only included in the correlation analyses and not in the latent growth curve models, as the primary focus was on paternal experiences during the peripartum period in relation to the risk of PB. This represents a limitation, as accumulating evidence suggests that maternal and paternal depressive, anxiety, and stress symptoms are interdependent [19,56–58]. Since mothers often spend more time in the NICU and experience immediate trauma, maternal postpartum depression is a strong predictor of paternal anxiety and depression [59]. A meta-analysis revealed that paternal depressive symptoms during the perinatal period are associated with maternal depressive symptoms at a later time point [60]. Thus, incorporating maternal psychological distress into the LGCM, ideally within a dyadic framework, could have provided a more comprehensive understanding of the trajectories of paternal emotional distress and the reciprocal influences between parents.

Despite these limitations, our study presents several important novel aspects. First, we distinguished between two groups of (prospective) fathers at risk of a PB, i.e., RG-PB and RG-TB, a differentiation made here for the first time. Second, we followed the three groups of fathers from pregnancy to postpartum, providing insight into mental health trajectories across the peripartum period.

Our primary findings reveal that fathers facing the risk of PB, but ultimately experiencing a term birth, represent a potentially vulnerable group, particularly preceding birth. The scarcity of studies following fathers of preterm infants during pregnancy [e.g., [17]] makes our research particularly valuable for understanding peripartum symptom development related to both threatened and actual PB.

### Implications and future directions

Even when preterm birth (PB) does not occur, the threat of PB alone is associated with higher levels of psychological distress in expectant fathers. Our findings suggest that assessing fathers’ emotional distress when the threat of PB is identified, may help detect those at increased risk and facilitate timely and effective psychological support.

A growing body of evidence indicates that partners’ emotional states mutually influence one another [56,61–64]. Given these reciprocal influences between parents [19,56,57,60], as well as the reported associations between parental mental health and child development [e.g., 65], future studies should adopt dyadic approaches that examine both parents or parent–child relationships [66]. To further investigate reciprocal effects in the context of threatened or actual preterm birth, we are currently examining longitudinal mother–father dyads and parental interactions. Beyond dyadic analyses, future research should also consider broader family systems, such as triadic models including both parents and their child. Such approaches may provide a more comprehensive understanding of family dynamics, as studying dyadic interactions in isolation can lead to fragmented or misleading conclusions [67].

Our findings underscore the importance of recognizing father’s role in early perinatal care. Fathers confronted with threatened PB should be routinely included in psychological screening and support programs during pregnancy and the postpartum period. Supporting fathers not only benefits their own mental health but may also promote maternal well-being, healthier family functioning, and, ultimately, more favorable developmental outcomes for children. In line with Bowlby’s attachment theory [68], elevated parental distress may, interfere with sensitive caregiving and the development of secure parent–infant attachment, as distressed parents may be less able to respond consistently and sensitively to their infant’s cues.

## Conclusions

Both the experience of threatened PB and the actual occurrence of a PB can significantly influence fathers’ well-being during pregnancy. The mere threat of PB is associated with increased psychological distress, even if a PB does not occur. Our study differentiates between the effects of PB threat and actual PB, showing that distress in RG-PB fathers can be related to both perceived risk and birth outcome. In conclusion, our findings underscore the need for psychological screening and support for fathers during the peripartum period, addressing both the psychological impact of the perceived PB threat and challenges of an actual PB.
